# The promise of Synovial Joint-on-a-Chip in rheumatoid arthritis

**DOI:** 10.3389/fimmu.2024.1408501

**Published:** 2024-09-11

**Authors:** Xin Zhang, Rui Su, Hui Wang, Ruihe Wu, Yuxin Fan, Zexuan Bin, Chong Gao, Caihong Wang

**Affiliations:** ^1^ Department of Rheumatology, The Second Hospital of Shanxi Medical University, Taiyuan, Shanxi, China; ^2^ Shanxi Key Laboratory for Immunomicroecology, Taiyuan, Shanxi, China; ^3^ Shanxi Province Engineering Research Center of Precision Medicine for Rheumatology, Taiyuan, Shanxi, China; ^4^ Pathology, Joint Program in Transfusion Medicine, Brigham and Women’s Hospital/Children’s Hospital, Harvard Medical School, Boston, MA, United States

**Keywords:** rheumatoid arthritis, Organ-on-a-Chip, Synovial Joint-on-a-Chip, disease models, precision medicine, microphysiological systems

## Abstract

Rheumatoid arthritis (RA) affects millions of people worldwide, but there are limited drugs available to treat it, so acquiring a more comprehensive comprehension of the underlying reasons and mechanisms behind inflammation is crucial, as well as developing novel therapeutic approaches to manage it and mitigate or forestall associated harm. It is evident that current *in vitro* models cannot faithfully replicate all aspects of joint diseases, which makes them ineffective as tools for disease research and drug testing. Organ-on-a-chip (OoC) technology is an innovative platform that can mimic the microenvironment and physiological state of living tissues more realistically than traditional methods by simulating the spatial arrangement of cells and interorgan communication. This technology allows for the precise control of fluid flow, nutrient exchange, and the transmission of physicochemical signals, such as bioelectrical, mechanical stimulation and shear force. In addition, the integration of cutting-edge technologies like sensors, 3D printing, and artificial intelligence enhances the capabilities of these models. Here, we delve into OoC models with a particular focus on Synovial Joints-on-a-Chip, where we outline their structure and function, highlighting the potential of the model to advance our understanding of RA. We integrate the actual evidence regarding various OoC models and their possible integration for multisystem disease study in RA research for the first time and introduce the prospects and opportunities of the chip in RA etiology and pathological mechanism research, drug research, disease prevention and human precision medicine. Although many challenges remain, OoC holds great promise as an *in vitro* model that approaches physiology and dynamics.

## 1 Introduction

**Figure 1:**
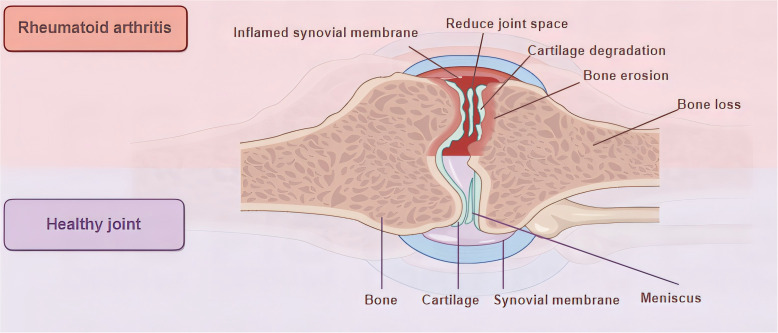
Joint characteristics of health and RA. During RA, the synovial membrane undergoes the following changes: infiltration of immune cells, new angiogenesis, uncontrolled proliferation of FLS, and the intima thickens and develops an aggressive pannus (6). Pannus tissue invades and destroys the underlying cartilage and bone (7), resulting in cartilage degeneration, bone destruction, and joint space narrowing.

**Figure 2:**
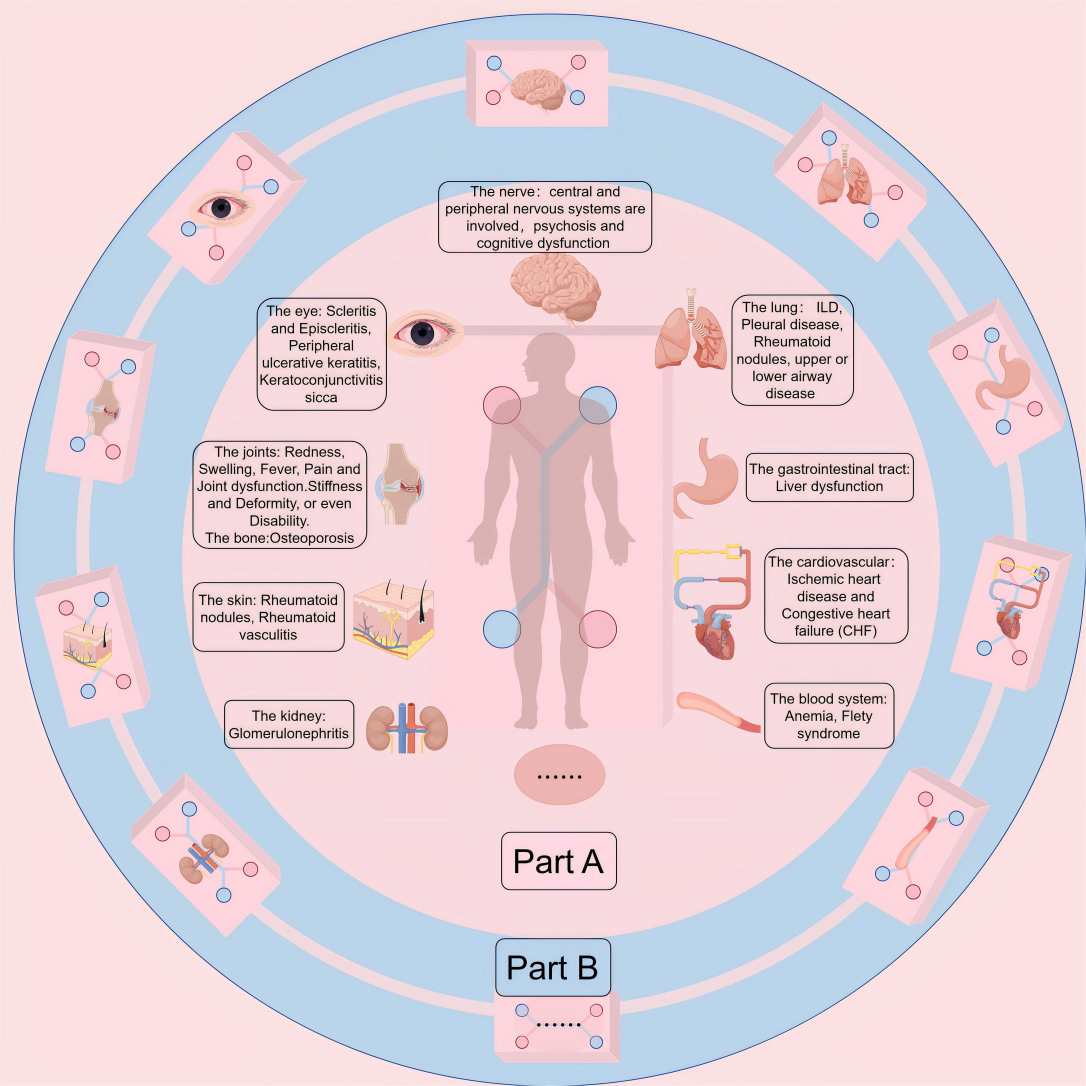
(Part A) Extra-Articular Manifestations (EMs) and comorbidities of RA. While synovitis serves as the pathological hallmark of RA, the intricate and persistent inflammatory and autoimmune nature of this disease gives rise to various Ems and comorbidities. These complications and comorbidities lead to increased morbidity and mortality. Among patients with RA, cardiovascular disease emerges as the primary cause of death, followed closely by respiratory ailments. (Part B) Multi-Organ-on-Chip (Multi-OoC) or Body-on-a-chip (BoC). OoC platforms can be interconnected to form more complex multi-OoC models or even BoC models, which can generalize interactions between various organs in the body, making it possible to study multi-tissue and even systemic diseases. Because intertissue crosstalk holds a pivotal position in the emergence and advancement of human, a common shared medium is often required for recycling that allows organ components to communicate with each other, while allowing them to retain their identity.

Rheumatoid arthritis (RA) manifests as an autoimmune disorder marked by persistent, aggressive arthritis. It affects about 0.5% to 1.0% of the global population and can occur at any age, with its highest incidence between 30-50 years, with the incidence of RA in women being 2-3 times higher than in men (1–4).The origins and development of RA remain intricate and a subject of ongoing research, with a prevailing belief that genetic, environmental, and autoimmune elements could be crucial in this condition (4, 5). Over time, these elements result in an overproduction of pro-inflammatory cytokines, represented by interleukin-6 (IL-6) and tumor necrosis factor (TNF), culminating in synovial cell proliferation, followed by cartilage destruction and bone erosion (4) (**Figure 1**). The primary clinical symptom of this illness is recurrent symmetrical polyarthritis, commonly seen in the hands, wrists, feet and other small joints (2, 8). In the initial stage of the disease, symptoms such as joint inflammation (redness, swelling, heat, pain) and joint disorders often occur (9). As the disease progresses, a range of different degrees of joint stiffness and deformity (10), or even disability (11), can be seen. However, since RA manifests as a systemic inflammatory condition, impacting not just joints but also various other extra-articular characteristics (4, 12, 13), such as skin, cardiovascular system, digestive system, respiratory system, etc. (14, 15) (**Figure 2** Part A). The current treatment methods for RA have low remission rate, many adverse reactions, and unsatisfactory efficacy, which seriously affect the quality of everyday living (16) and physical and mental health (17), and bring burdens to individuals and society (18–20). Therefore, it is essential to deepen our comprehension of the etiology and pathogenesis of RA and develop new and more effective drugs to alleviate the suffering of patients.

A wide range of *in vitro* models have been employed to reveal the tissue development and pathogenesis of joints. These include simple 2D monolayer cultures of fibroblast-like synoviocytes (FLS) (21, 22), as well as more complex models such as tissue explants of bone (23), cartilage (24, 25), synovium (26) and meniscus (27, 28), co-culture of cartilage and synovium (29) and multi-compartment bioreactor (30, 31), among others. Despite their contributions, traditional *in vitro* models have limitations when it comes to accurately reflecting physiological conditions (**Table 1**). Similarly, animal models (such as mice), which are a vital part of RA research, suffer from species-specific differences (32, 33) and high costs (34, 35). Consequently, it’s critically important to create innovative, dynamic, and physiological 3D cell culture models as substitutes for additional studies.

**Table 1:** Existing models for studying joints.

Models		Major Advantages	Major Disadvantages
2D Monolayer culture		·High-throughput ·Simple and easy to work	·Lack of mechanical stimulation ·Fail to replicate the complex cell–cell and matrix–cell interactions ·The altered phenotype of cells
3D cell-based	Co-culture of cells	·Reproduction of intercellular and cell-matrix interactions	·Enable to mimic only one tissue type
	Bioreactor	·Extended culture time and quality of explants/tissues	·No tissue-tissue interface and chemical concentration gradient
	Tissue explant	·Reflecting the human physiology in terms of 3D structure and environment	·Difficult to collect and preserve ·Lack of reproducibility
	Organoid	·Recapitulates native organ architecture and cell types.	·There are differences in size and human body, and the function is limited ·Lack of neural, immune and circulatory structures
3D Scaffold-based or 3D printed-based		·Scaffold mechanically supports cells and promotes cell survival, proliferation, migration and differentiation· ·3D Bioprinting offers low-cost production, digital control of highly resolution patterns and high printing speed	·No control of parameters at the single-cell level
OoC		·Mimic the physiological microenvironment of the *in vivo* target organs faithfully. ·Provide tissue mechanical force and a controllable microenvironment ·Highspeed, parallel collection, and analysis of individual biological information.	·Difficult to standardize ·High complexity and technical requirements
Animal models		·The repeatability was good	·Species-specific differences ·Ethical issues

Compared with the traditional *in vitro* cell models, OoC enable precise control over cell cultivation (36), cell spatial configuration (37), and inter-organ interactions (38). Unlike static organoid culture, OoC technology enables the efficient movement of fluids, thereby improving nutrient transfer and the conveyance of physicochemical cues like bioelectrical stimulation (39), mechanical stimulation (40), and shear force. Consequently, this method provides a more precise depiction of the physiological states present in living tissue (41). Moreover, the use of OoC technology in physiological barrier models accurately mimics the delivery and penetration of compounds *in vivo (* 42). In recent years, there has been a notable enhancement in the precision of OoC, enabling single-cell detection and high-throughput capabilities (43).

In this review, we outline the structural features and benefits of OoC, highlighting its advantages over traditional models in terms of replicating the complexity of biological systems. We then provide an overview of the current applications and accomplishments of the Synovial Joint-on-a-Chip (JoC), a specialized OoC model designed to mimic the human synovial joint. We describe the fundamental structure and functions of JoC, emphasizing its relevance to the study of RA. In addition, we delve into the potential applications of JoC in RA research, including its utility in investigating the underlying causes of the disease, understanding its pathological mechanisms and aiding drug development, and how JoC can contribute to disease prevention strategies and the field of precision medicine. We believe that although JoC still faces many challenges, with more in-depth research and the development of new technologies, the application of this chip in disease research, especially precision medicine, will be further promoted.

## 2 Organ-on-a-Chip: a novel and promising *in vitro* model

Organ-on-a-Chip (OoC, namely, microphysiological systems) is a micrometer-sized cell culture device that integrates microfluidic technology, biomaterials, and cell biology (6, 7), aiming to replicate the basic structural and functional characteristics of a specific tissue or organ (44–47). The creation of OoC systems is dependent on four key elements: First, microfluidics plays a crucial role in delivering nutrients and maintaining cellular homeostasis while also facilitating the removal of waste products. Second, living cells are integrated with biomaterials, such as hydrogels, to create a supportive environment that mimics the natural extracellular matrix. Third, physicochemical stimuli are applied to simulate the *in vivo* microenvironment, enhancing the system’s ability to replicate physiological conditions. Finally, sensors are incorporated to collect, process, and analyze data and images, providing valuable insights into cellular behavior and responses (7). Traditionally, microfluidic culture devices have been manufactured using soft lithography technology with materials like polydimethylsiloxane (PDMS). However, 3D printing has become a promising technology for microfluidic devices due to its low cost, standardization, and rapid production (48–50). In general, the corresponding OoC is constructed by simplifying and analyzing the different cell types and organ-specific microenvironments of the target organ and combining with the research objectives.

First and foremost, it is important to understand the target organ to determine the microstructure, including the biological context, size, and geometry-whether the microchamber or microchannel (single or multiple, parallel or sandwich structure). For example, a Synovial Membrane-on-a-Chip needs to be designed as a sandwich structure containing upper and lower layers separated by a membrane that supports intercellular communication. However, cartilage microarray usually only requires the design of a culture chamber to accommodate chondrocytes. The selection of appropriate cells is a critical step in the development of an OoC system. Cells are derived from primary human cells or mature, well-characterized cell lines (51), and stem cells, especially induced pluripotent stem cells (iPSC) (52), are an area of active and potential research. To create an environment that closely resembles the natural extracellular matrix (ECM), cells are often embedded within hydrogels. These materials can be synthetic, such as polyethylene glycol (PEG), or naturally derived, including agarose (53), alginates (54), or polysaccharides such as hyaluronic acid and glucan (55). The choice of hydrogel depends on the specific needs of the model and the desired mechanical and biochemical properties. The integration of cells within these hydrogels and their subsequent injection into the microstructures form the basis of the OoC system. This approach allows for the creation of a microenvironment that supports cell growth, function, and interaction, providing a powerful tool for studying organ-specific diseases such as RA.

Then, the integrity of OOC was increased depending on the environment to which the cells were exposed and the stimuli received. For instance, in an alveolar-capillary OoC model, alveolar epithelial cells and pulmonary microvascular endothelial cells are exposed to their respective tissue-specific environments, with air on the alveolar side and fluid on the vascular side, thus mimicking the natural conditions each cell type experiences (56). To simulate the mechanical forces that cells experience in the body, innovative design solutions have been employed. For example, vacuum pumps have been used to apply traction and compression forces to PDMS membranes, deforming them in a controlled manner (57). In the Intestinal-on-a-Chip, epithelial cells under drip and periodic mechanical deformation. In the study of musculoskeletal systems, increasing mechanical stimulation is essential to mimic the impact of physical activity on cells. Similarly, for tissues with significant electrical activity, such as those in the nervous system and the heart, integrating electrical stimulation into OoC systems is vital to enhance their physiological relevance. In addition, almost all cells are affected by biological chemical stimulus such as cytokines, growth factors, and signaling molecules, which play crucial roles in physiological responses, tissue development, and intercellular communication. Therefore, incorporating these factors into OoC models is crucial for accurately reflecting cellular behavior. Additionally, physical environmental conditions such as temperature, pH, and oxygen concentration must be carefully controlled to optimize the performance and quality of OoC systems, ensuring that they provide a reliable and representative model of the organ or tissue being studied.

In the past, the measurement of relevant parameters in the OoC platform could be achieved by multi-electrode matrix (MEA), transepithelial electrical resistance (TEER) and enzyme immunosorbent assay (ELISA) (40, 58, 59). However, the need to continuously monitor the environment and cellular behavior in real time has driven the inclusion of sensors. Such as measurements of plasma cytokines sensors (60), used to measure oxygen concentration colorimetric or fluorescence sensors (61) and electrochemical microsensors to monitor pH (62), oxygen concentration (63), and cell metabolism (64), etc.

OoC have been employed to simulate a wide range of functional tissues or organs, encompassing the lung (39, 57, 65–67), liver (68–71), heart (72–74), gut (75–77), kidney (78, 79), brain (80, 81), skin (82–84), and tumors (85–88),among others. Interlinking various OoC platforms can lead to the creation of intricate multi-OoC models (89, 90) or even Boc models(**Figure 2 Part B**) (91–94), facilitating the exploration of diseases affecting multiple systems or systemic diseases. RA manifests as a systemic condition that can accumulate almost every organ in the body, and people are very interested in the multi-OoC and even the BoC model that simulates RA. OoC devices have emerged as the forthcoming wave of *in vitro* models, bridging the divide between conventional preclinical *in vitro* models and clinical experiments (95).

## 3 Synovial Joint-on-a-Chip

The joint consists of a complex network of tissues, including various tissues such as articular cartilage, subchondral bone, synovial membrane, ligaments, and meniscus, as well as auxiliary tissues, including Hoffa fat pads, muscles, tendons, and knee patella, each with specific structural characteristics and functions. A JoC close to the physiological state should contain all parts of the joint structure, but in most cases, people add only the required parts appropriately according to their own research purposes (**Table 2**). Different cell types and compositions as well as complex tissue structures and interactions present a significant challenge in creating *in vitro* models that accurately represent the microenvironments of joint tissues. Therefore, creating innovative biological models for human joints to enhance the study of joint physiology and pathology, like osteoarthritis (OA) and RA, is essential yet challenging.

**Table 2:** Currently developed JoC models.

Joint Models	Cells/Tissues type	Main Findings/Uses	References
A human 3D chip of RA	FLS, Chondrocytes	• First established a chip-based chondro-synovial dual organoid model, allowing the study of mutual tissue-tissue cross talk in arthritis research. • Co-cultivation of chondral and synovial organoids improved the phenotype of chondrocytes within chondral organoids in comparison to chondral monocultures.	(96)
A cartilage-on-a-chip model of RA	Human monocyte cell line (THP-1), Human chondrogenic primary cells (hCH)	The developed preclinical model allowed us to provide more robust data on the potential therapeutic effect of anti-TNF a mAb-CS/PAMAM dendrimer NPs loaded-Ty-GG hydrogel in a physiologically relevant model.	(97)
Human vascularized synovium-on-a-chip	FLS, Human umbilical vein endothelial cells (HUVECs).	• Inflammatory markers in blood vessel channel enhanced with circulating monocyte adhesion to increase. • This vascularized human synovial fluid sheet model recapitulates many functional features of healthy and inflamed human synovial fluid to understand synovial joint disease mechanisms, allows identification of novel therapeutic targets and supports preclinical testing of therapies.	(98)
3D chondrocyte culture-on-a-chip of OA	Chondrocytes	The results show that our microtissue model mimics the essential features of native cartilage and can respond to biochemical injury, thus providing a new basis for exploring the pathophysiology of osteoarthritis.	(99)
A microfluidic chip of RA	FLS, Osteoclastic, Bone marrow mesenchymal stem cell (BMSC)	• Simulated FLS migration and invasion mediated bone erosion in RA. • RA positive drug celastrol inhibition of FLS cell migration and tartrate resistant acid phosphatase (TRAP) activities.	(100)
A joint mimicking loading system	Chondrocytes	• To investigate the effect of combined stimulation on the zonal organization of cartilage. • Mechanical loads applied in joints play a crucial role in stimulating ECM production and its functional rearrangement.	(101)
Monolithic microfluidic platform of OA	Chondrocytes	• The PDMS membrane of the device allows mechanical stimulation of the chondrocytes. • Chondrocytes cultured close to the side of high physiological stimulation had lower viability.	(102)
A cartilage-on-a-chip model of OA	Chondrocytes	• Hyperphysiological compression in this model triggers the transition of cartilage homeostasis to chondritis and inflammation and hypertrophy, and a gene expression profile similar to that seen in clinical OA tissue can be obtained, enabling the screening of DMOA candidates.	(103)
Osteochondral Tissue Chip of OA	IPSCs differentiate into cartilage and osteoblasts	Celecoxib, a prescription drug for OA, downregulated the expression of catabolic and proinflammatory cytokines in this model, demonstrating the utility of this model for drug screening.	(104)
Organotypic microfluidic model of OA	Chondrocytes, FLS, Synovial fluid	The platform is designed to study the biological mechanism of monocyte extravasation in synovial membrane and will be used in the future to test compounds that target the chemokine signaling axis responsible for monocyte recruitment.	(105)
Miniature Joint System of OA	Osteoblasts, Chondrocytes, FLS, Adipocytes	The potential of miniJoint to predict the *in vivo* efficacy of drug therapies was demonstrated, thus providing a powerful OoC model for the study of joint pathology and the development of novel therapeutic interventions.	(106)
A miniature knee joint system (known as the miniJoint) of OA	Osteoblasts, Chondrocytes, FLS, Adipocytes	• The combined treatment of bone morphogenic protein-7(BMP-7) and oligodeoxynucleotides reduced inflammation in the synovial-like fibrous tissue and increased the formation of glycosaminoglycan in the cartilage fraction. • This is the first demonstration of the potential of micro-joints to develop drugs to improve OA disease.	(107)
A cartilage-on-chip	Chondrocytes	This model allows multiple experimental parameters to be studied, opening new avenues for basic research in cartilage tissue and drug testing research in arthritic diseases.	(108)
Microphysiological Osteochondral System of OA	Human bone marrow stem cell (HBMSCs) differentiated into upper cartilage-like tissue and lower bone-like tissues.	This model provides novel capabilities for the physiology of osteochondral tissue and the pathogenic mechanisms of OA, and serves as a high-throughput platform to test potential DMOADS	(30)
A 3D synovium-on-a-chip	FLS	The incorporation of intricate human synovial organ cultures into a lab-on-a-chip platform yields consistent and dependable insights into the impact of systemic stress factors on synovial tissue structures.	(109)
3D osteochondral microtissue of OA	Bone, Osteochondral interface, Cartilage, Synovium,	It will eventually serve as an improved, high-throughput *in vitro* model for predicting the efficacy, safety, bioavailability, and toxicological outcomes of candidate OA drugs.	(31)

### 3.1 Osteochondral-on-a-Chip

Articular cartilage is a vascular and nerveless structure composed mainly of chondrocytes (110). Collagen fibers and glycosaminoglycans (GAG) are important extracellular matrix components (111). Different from cartilage, the subchondral bone is a highly vascularized and innervated tissue, characterized by its hard, calcified matrix made of type I collagen and calcium phosphate (hydroxyapatite). Physiological maintenance of bone homeostasis hinges on the equilibrium of bone development by osteoblasts and bone degradation by osteoclasts (112, 113). Calcified cartilage in the center divides the articular cartilage from the subchondral bone. Cartilage behavior is shaped by the architecture and composition of the subchondral bone, and there is molecular and physical crosstalk between the two types of tissue tissues (110, 114). In order to establish an accurate human Synovial JoC model to study the interactions between tissues, the combination of articular cartilage, osteochondral interface, and subchondral bone into an engineered Osteochondral-on-a-Chip has an important role in clarifying the development of joint ailments and assessing the effectiveness of possible treatments (31).

**Figure 3:**
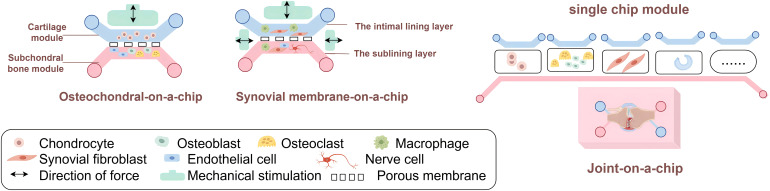
Synovial Joint-on-a-Chip. The multi-tissue characteristic of joint determines the complexity of synovial joint-on-a-chip. When making JoC, the usual method is to separate each joint tissue into a single chip module, and then connect the required part appropriately according to their own research purposes.

The Osteochondral-on-a-Chip is usually designed as a device of two compartments (**Figure 3**). Each compartment is composed of a suitable hydrogel matrix containing different cells (one compartment is chondrocytes, simulating articular cartilage; The other compartment is used to simulate subchondral bone, containing osteoblasts, osteoclasts and endothelial cells). It’s essential that the two hydrogel matrices possess varying hardness levels to effectively facilitate tissue formation and promote vascularization in specific areas. A thin, permeable porous membrane can divide these compartments, aiding in the communication between bone and cartilage. Furthermore, each of the two compartments will experience mechanical forces.

The Osteochondral-on-a-Chip of this structure have been reported several times. Research delves into how mechanical damage, contact with inflammatory cytokines, and weakened bone integrity influence the deterioration of cartilage in the osteochondral microsystem (31). Lin et al. (104) simulated the pathology of OA with microphysiological osteochondral tissue microchips and observed that celecoxib, a selective COX-2 inhibitor, down-regulated the levels of both pro-inflammatory cytokines and catabolic in the OA model, highlighting the potential usefulness of Osteochondral-on-a-Chip in drug testing. A similar chip mimics the tissue response to interleukin-1β (30).

### 3.2 Synovial Membrane-on-a-Chip

The synovial membrane consists of the intimal lining layer facing the joint cavity and the sublining layer. The intimal lining layer is home to two types of cells—macrophage-like synoviocytes (Type A cells) and fibroblast-like synoviocytes (FLS, Type B cells) (115–117). These loose structural features, on the one hand, allow the diffusion of nutrients to feed the avascular cartilage, and on the other, may lead to the accumulation of inflammatory substances such as immune complexes within the joint. The lamina propria contains synovial cells, macrophages, nerves and microvessels. The synovial fluid, rich in proteins, plasma, and lubricants like hyaluronic acid produced by FLS, plays a crucial role in joint nourishment and lubrication.

Synovial Membrane-on-a-Chip (also known as Synovium-on-a-Chip) is typically implemented through two microfluidic chamber devices separated by a thin membrane that support cell-to-cell communication. The upper chamber contains a layer of FLS or FLS and macrophages, while the lower chamber contains embedded FLS, macrophages, endothelial cells and nerve cells, all of which are embedded in the 3D hydrogel (**Figure 3**). To replicate the effects of shear stress caused by joint fluid during movement and blood circulation, either partial or ongoing perfusion in both chambers can be employed. Where membrane stretching is used, for example, in Lung-on-a-Chip (57) devices to simulate breathing and Intestine-on-a-Chip (118) devices to simulate peristalsis.

A variety of OoC systems have been created to investigate synovium’s function in rheumatic diseases. To illustrate, a vascularized Synovium-on-a-Chip that incorporates mechanical stimulation was reported to study healthy synovial membranes, synovial inflammation, and its effects on circulating monocyte behavior (98). A JoC model including vascularized synovial membrane and articular cartilage was developed and validated in a study to investigate the exfiltration of monocytes into the synovial membrane (119). A microfluidic chip-based co-culture system was evaluated for its ability to mimic bone erosion caused by FLS in RA and its potential for drug testing (100). Rothbauer and his team engineered a 3D Synovium-on-a-Chip setup to track the emergence and development of inflammatory responses in synovial tissues (109). For closer physiological proximity, the nearest Synovial Membrane-on-a-Chip incorporates synovial fluid (120).

### 3.3 Other joint tissues chips

Other joint tissues, including tendons, ligaments, meniscus, and Hoffa fat, we can envision designing these tissue chips in a plug-and-play form that can be flexibly adapted to applications driven by clinical needs. Lymphatic vessels have been incorporated into *in vitro* models of RA in several studies (121).When making the decision on which tissues or components to incorporate, it is important to find a middle ground between complexity and accuracy. The goal is to maintain a JoC model that remains biologically and/or physiologically relevant, while also promoting its widespread adoption through simplicity and user-friendliness.

### 3.4 Sensors for RA

Has now developed a variety of biological sensors used in RA. Some optical biosensors are used to detect RA-related biomarkers, including microRNAs (miRNAs), anti-citrullinated protein antibodies (ACPA) (122, 123), rheumatoid factor (RF) (124), and C-reactive protein (CRP) (125–127). Electrochemical nanosensor for the detection of ACPA (128). A sandwich dual electrochemical biosensor for the simultaneous detection of anti-cyclic citrullinated peptide (CCPA) and RF autoantibodies with high sensitivity and efficiency was recently reported (129). Lin et al. developed a peptide electrochemical sensor based on electrochemical impedance spectroscopy, for detecting RA autoantibodies (130). Due to the small size of nanomaterials and their unique electronic, physical and chemical properties (131), nanobiosensor devices have been developed for RA (132, 133) and will become a vital component of the next generation of point-of-care diagnostic tools, offering rapid and accurate testing options (134).

## 4 Prospects and opportunities of Synovial Joint-on-a-Chip in RA

### 4.1 To investigate the etiology and pathogenesis of RA

The onset and development of RA is linked to a range of different factors, including genetics, immunity, and the environment (such as smoking). A key benefit of using OoC *in vitro* models lies in their capacity to examine a single factor sequentially, thereby reducing potential disruptions in live studies (95). With the support of microfluidic technology, Several OoC devices, designed to mimic the etiology of diseases, have been created. A 3D Synovium-on-a-Chip system with four individual microcompartments has been developed to monitor the occurrence and progression of inflammatory synovial tissue responses (109).

#### 4.1.1 Genetic susceptibility

Recent research has uncovered an expanding array of genetic factors associated with joint disorders, including epigenetic changes and genomic variations (135). Genetic factors stand out as the primary risk factor for RA (1). It is feasible to conceive of two main applications of synovial articular microchips in unraveling RA genetic susceptibility. Not only can we study specific gene functions by creating “genetically modified” OoC, but the utilization of comparative transcriptomics in the analysis of tissues derived from OoC models generated by cells from various patients and disease subgroups has the potential to elucidate previously unidentified genetic inclinations to joint diseases (135). We have reason to believe that in the near future, this new model will also be used in RA etiology and pathogenesis research.

#### 4.1.2 Environmental factors

Smoking is known to significantly escalate the danger of RA. Benam et al. (136) constructed a smoking airway chip composed of four integrated components *in vitro* to study the smoke-induced pathophysiology. Another study connected small airway chips to biomimetic smoking robot microfluidics to investigate how e-cigarettes impact genetic, molecular, cellular, and tissue reactions in human lungs under controlled conditions (137). The development of cigarette chips, trachea chips, and lung chips is expected to further the understanding of how smoking affects RA.

#### 4.1.3 Immune disorder

The immune system is pivotal in the emergence and advancement of numerous rheumatic conditions (138), and since immune malfunction is a key factor in the development of RA, it ought to be incorporated into the suggested JoC model. Recent extensive research has shown that changes in the gut microbiota of RA patients significantly contribute to the development of abnormal systemic immunity (139–141). Lately, the rapid evolution of Gut-on-a-Chip (or Intestinal-on-a-Chip) technology has opened new avenues for disease research, including RA. 1) Gut-Microbiome-on-a-Chip. The Gut-on-a-Chip developed by Jalili-Firoozinezhad’s team coculture human gut epithelial cells with either anaerobic or aerobic gut microbiota (by producing a controlled oxygen gradient) to examine the immediate connections between gut bacteria and intestinal tissues (142).A separate research by Gumuscu and colleagues involved co-culturing intestinal bacteria (*E. coli*) cells with Caco-2, perfusion continuously using a microfluidic apparatus, to initially assess the effects of drugs (143). 2) Gut-Immune Interactions-on-a-Chip. Kim and colleagues have created a bespoke human Gut-on-a-Chip system designed to explore the dynamics of gut and immune responses (144). Similarly, Shin and Kim designed an immune model using a similar approach to observe the early stages of inflammation in the gut (145).

Currently, the Gut-on-a-Chip is already used for conditions such as phenylketonuria (146), viral infections (147) and inflammatory bowel disease (IBD) (148). Although there is no precedent for RA, the gut is closely related to RA (intestinal infection can be the cause of RA, intestinal microbial disturbance is the pathogenesis of RA, RA can be manifested as digestive system involvement, and the gut itself and intestinal flora have a certain effect on drug efficacy). The growing research on the gut-joint axis suggests a promising future for Gut-on-a-Chip technology in the study of RA.

### 4.2 Screening of drugs

Developing new therapeutic agents and treatments is a recognized challenge due to its high cost, complexity, extended duration, and high failure rates. The OoC technology, with its high throughput, integration, and reproducibility, offers a promising solution to reduce the costs associated with drug research and development and is increasingly being utilized in drug screening and analysis.

#### 4.2.1 Modeling of pharmacokinetics and pharmacodynamics

Following the identification of potential molecules and targets, studies focusing on pharmacokinetics and pharmacodynamics (PK and PD) are conducted. On the one hand, PK is used to describe the metabolic process of drug candidates *in vivo*, namely absorption, distribution, metabolism and elimination (ADME). On the other hand, PD refers to the drug’s impact on a specific tissue or organ, namely, how pharmacology or toxicology interacts with the drug’s dosage or concentration (149). Integrating PK/PD factors is vital for the advancement of new medications, as it forecasts potential drug reactions, thereby minimizing harmful metabolites and side effects (93, 150).

#### 4.2.2 Evaluating drug safety and efficacy

The safety and efficacy of drugs are usually not predicted in animal models (151–153).The pharmacokinetics of drugs are affected by many factors, and toxicity testing and screening of drugs with inappropriate disease models may lead to potential multi-organ side effects (41). Multi-OoC (154) or human BoC models (94), which couple complex whole-body physiological reactions from two or more organ chips *in vitro*, facilitate the study of organ interactions and the identification of possible adverse effects (155). Additionally, these models offer a basis for examining interactions between drugs (156). A microfluidic chip co-culture of FLSs with osteoblasts and osteoclasts reestablishes the migration and invasion abilities of bone-associated cells, providing a valuable tool for anti-RA drug screening for FLS migration-mediated targeted bone erosion (100).

Furthermore, during the quest for novel medications, an additional critical factor impacting drug efficacy is the effective method of drug delivery within the patient’s system (157). Therefore, developments in Skin-on-a-Chip for transdermal administration assessment (158), Lung-on-a-Chip for inhalation administration evaluation (159, 160), Gut-on-a-Chip for oral/rectal administration testing (161, 162), and models for vascularization in intravenous administration testing (163) have been achieved. Additionally, this approach facilitates the examination of potential drugs in at-risk groups such as expectant mothers, kids, and the aged, who are frequently omitted from clinical studies (164).

#### 4.2.3 Personalized drug therapy

In recent years, high-throughput OoC utilizing multi-chamber and compatible dosing concentration gradient generators is a new method for drug development or “personalized” drug therapy, which can automatically process multiple drug combinations of different concentrations within a brief span of time to improve the efficiency of drug development (165). The organoid chip developed by Schuster et al. (166) can perform individual, combined and ordered drug screening.

The continuous or sampling drug administration, coupled with managing the culture environment, enables the evaluation of intricate dosing doses and cycles, and even combined therapies. This will provide a more precise and personalized regimen for the periodic combination of medications in RA patients. Evidence has shown that the anti-inflammatory and anti-metabolic responses of certain drugs [Celecoxib, a drug that is non-steroidal and anti-inflammatory (167), cortisol hormone dexamethasone (168), interleukin-1 receptor antagonist (169)] can be predicted using in a Cartilage-on-a-Chip model (103). OoC, especially multi-OoC, a new *in vitro* model, is expected to screen potentially effective drugs for improving RA symptoms and controlling disease progression, and even take into account the functional status of patients’ organs to achieve personalized medication.

### 4.3 Diagnosis and early prevention

The progression of RA initiates in a state of health, succeeded by the onset of preclinical RA, which poses a risk for RA, then transitions to early synovitis, and ultimately culminates in established, destructive disease (1). According to the 2010 American College of Rheumatology/European League Against Rheumatism (ACR/EULAR), early diagnosis and treatment are important in improving the prognosis of RA. The detection method that simultaneously targets RF and anti-CCP is integrated in a 45 mm × 62 mm microfluidic chip, which is of great significance for the diagnosis of RA, especially early RA (170). Studies have shown that depending on the cells and stimuli used, OoC can be used to model the different stages of RA development (i.e., preclinical RA, early RA, and established RA) to understand the impact of RA pathology on various organs over both immediate and extended periods (171), which could lead to renewed optimism in the early detection and potential prevention of RA. However, at present, joint tissues are mainly obtained from patients with advanced joint diseases (172). Lack of joint tissue in healthy or early-stage joint disease is a major obstacle.

### 4.4 Individual precision medicine

Individuals exhibit unique genetic profiles, diverse living conditions, and varied disease mechanisms, leading to distinct clinical presentations and complications. Tailoring medical care to these individual differences is essential. It is meaningful to stratify patient subgroups and even create “Your Chip” or “Patient-on-a-Chip” to achieve true personalized medical care. Even within the same patient, changes in the chip’s responses at different disease stages can provide valuable insights for targeted interventions. OoC applications may be particularly useful in cases where alternative techniques fail to replicate genetic disorders (173), or when studies in humans are difficult (e.g., special populations such as pregnant women and young children, new drugs that have not yet been tested in clinical trials, potentially pathogenic radiation, etc.).

Despite the rapid development of OoC technology, integrating various mature tissues while maintaining their characteristics in multi-OoC systems is still in its infancy. Phenotypes during organ maturation are influenced by a variety of factors, such as the microenvironment composed of nutrients, metabolites, local substrates, circulating immune cells, and metabolites. As a result, the BoC has not yet been fully realized, although it is developing at a rapid pace.

## 5 Conclusion and future prospects

In this review, for the first time, we have compiled practical evidence that various systems are establishing OoC, and innovatively analyzed the possibility of integrating various systems into systemic disease RA. This move undoubtedly provides a new alternative model for studying RA. Although there are still challenges and shortcomings, OoC combines the advantages of medical biology and engineering, and can effectively solve the constraints of animal models and traditional *in vitro* models. In addition to realizing 3D cell culture, the system reproduces the relationship between cells and their matrix. Moreover, it can simulate the interstitial fluid and mechanical force stimulation of target organs *in vivo*, which has unparalleled advantages in the bionic microenvironment.

The future development trend of OoC will be a system composed of multiple organs with the help of connected pipes-human organ bionic chips. This requires more research on population proportions, differentiation conditions, spatial distribution among multicellular populations, and the determination of amplification factors to simulate the complex physiological environment *in vivo*. Due to its reproducibility and accuracy, OoC is likely to completely change the way medical research is conducted in the future, replacing animal and 2D cell experiments and becoming a new tool for more functional, integrated, automated and personalized preclinical research.

The utilization of emerging technologies, such as artificial intelligence and regulatory algorithms, to establish connections with the OoC systems has the potential to enhance their application in the fields of precision medicine and systems biology (174). With the breakthrough of engineering challenges, the integration of technologies in various fields, and the smooth advancement of the industrialization layout of corresponding companies, emerging technologies such as 3D printing, OoC and nanotechnology may be expected to trigger a revolution in the creation of novel medications and make the track full of vitality.
